# Recording and Analysis of Bowel Sounds

**DOI:** 10.5005/jp-journals-10018-1137

**Published:** 2016-07-09

**Authors:** Daniel Zaborski, Miroslaw Halczak, Wilhelm Grzesiak, Andrzej Modrzejewski

**Affiliations:** 1 Laboratory of Biostatistics, West Pomeranian University of Technology, Zachodniopomorskie, Poland; 2 Department of Surgical and Emergency Nursing, Pomeranian Medical University, Zachodniopomorskie, Poland

**Keywords:** Appendicitis, Ileus, Intestinal motility, Peritonitis.

## Abstract

**Background:**

The aim of this study was to construct an electronic bowel sound recording system and determine its usefulness for the diagnosis of appendicitis, mechanical ileus and diffuse peritonitis.

**Materials and methods:**

A group of 67 subjects aged 17 to 88 years including 15 controls was examined. Bowel sounds were recorded using an electret microphone placed on the right side of the hypogastrium and connected to a laptop computer. The method of adjustable grids (converted into binary matrices) was used for bowel sounds analysis.

**Results:**

Significantly, fewer (p ≤ 0.05) sounds were found in the mechanical ileus (1004.4) and diffuse peritonitis (466.3) groups than in the controls (2179.3). After superimposing adjustable binary matrices on combined sounds (interval between sounds <0.01 s), significant relationships (p ≤ 0.05) were found between particular positions in the matrices (row-column) and the patient groups. These included the A1_T1 and A1_T2 positions and mechanical ileus as well as the A1_T2 and A1_T4 positions and appendicitis. For diffuse peritonitis, significant positions were A5_T4 and A1_T4.

**Conclusion:**

Differences were noted in the number of sounds and binary matrices in the groups of patients with acute abdominal diseases. Certain features of bowel sounds characteristic of individual abdominal diseases were indicated.

**List of abbreviations:**

BS: bowel sound; APP: appendicitis; IL: mechanical ileus; PE: diffuse peritonitis; CG: control group; NSI: number of sound impulses; NCI: number of combined sound impulses; MBS: mean bit-similarity; TMIN: minimum time between impulses; TMAX: maximum time between impulses; TMEAN: mean time between impulses.

**How to cite this article:**

Zaborski D, Halczak M, Grzesiak W, Modrzejewski A. Recording and Analysis of Bowel Sounds. Euroasian J Hepato-Gastroenterol 2015;5(2):67-73.

## BACKGROUND

Digestive tract motility consists in the contraction of the circular or longitudinal layer of the smooth muscles and the change in sphincter tone. It is one of the elements that enable food digestion and absorption of its products. In the small intestine, segmental and peristaltic contractions can be distinguished.^1^ In the large intestine, also mass contractions occur, which propel stool into the rectum.

Intestinal motility depends on many factors, such as the amount and type of ingested food, emotional state, drugs,^2^ disease entities and operative procedures. The peristalsis of the digestive tract is accompanied by sounds^3-5^ whose source is the passing mixture of chyme and gas.^6^ Clinical examination includes the auscultation of peristaltic sounds using a stethoscope.^7^ The character of bowel sounds (BS) altered in acute abdominal diseases and after operative procedures is an important diagnostic indicator. A significant increase in BS intensity takes place in the onset of mechanical ileus, food poisoning with diarrhea and gastrointestinal hemorrhage. The complete absence of these sounds indicates complete paralytic ileus and occurs mainly in the diffuse peritonitis, appendicular perforation, peptic ulcer perforation, in the final phase of mechanical ileus and in intraperitoneal bleeding. Operative procedures, in which the peritoneal cavity is opened, result in the temporary intestinal paralysis and, consequently, the absence of BS.^7-10^

Bowel sounds auscultation, commonly applied in clinical practice, is based on subjective assessment using the stethoscope. The most frequently described parameters are sound frequency and loudness. It can be assumed that BS provides much more information on disease or health, which could be used in diagnostics.

Therefore, the aim of the present study was: (1) to construct a device for BS recording, (2) to develop the methods for saving and storing data, (3) to determine the characteristic features of BS from healthy subjects and patients and their usefulness in the diagnosis of acute abdominal diseases.

## MATERIALS AND METHODS

Bowel sounds were recorded using a standard electret microphone (placed in a Teflon profiled sleeve), a two-stage voltage amplifier and a laptop computer equipped with a 16-bit Sound Blaster card. Headphones with an amplifier were also used to control recording process. Four-minute recordings were saved to the computer hard disk as wave files with a sampling frequency of 16 kHz.

Bowel sounds were auscultated after obtaining patient’s informed consent. The study was approved by the local ethics committee. The patient was laid supine and the microphone was placed on the abdomen skin, 2 to 3 cm to the right of the umbilicus. In the case of excessive hair, the microphone was moved in the lateral direction. During the examination, the patient was requested to remain silent, avoid making any movements and breathe calmly. The sounds were recorded in a quiet isolated room. The quality of the recorded signal was being controlled in real time using the headphone system. The examinations were performed in 67 subjects after an overnight fasting hospitalized in the third clinic of general and vascular surgery. Seven persons were excluded from further analysis due to the low quality of recordings.

Patients’ age ranged between 17 and 88 years (mean 48.3 years). Women were in majority (63%). Forty-five subjects with acute abdominal diseases were examined. Control group (CG) included patients hospitalized in the clinic due to other diseases. Bowel sounds in the subjects with the symptoms of acute abdomen were recorded after preliminary diagnosis and before surgical procedure. Four groups of patients were distinguished.

The group of 27 patients with appendicitis (APP) included 18 women, 17 to 74 years of age (mean age of 32.9 years) and 9 men, 17 to 71 years of age (mean age of 37.1 year). In two cases, appendicitis was accompanied by the ovarian cyst rupture, in one case by Meckel’s diverticulitis and also in one case by the sigmoid perforation. Among 14 patients with mechanical ileus (IL), there were eight women aged 42 to 88 years (mean age of 68 years) and six men aged 56 to 76 years (mean age of 66.5 years). Most frequently (in six cases), mechanical ileus was caused by peritoneal adhesions, followed by incarcerated hernia (four patients), neoplastic disease (two patients), intussusception (one patient) and sigmoid volvulus (one patient). In the group of four patients with diffuse peritonitis (PE), there was one 84-year-old woman and three men 40 to 53 years of age (mean age of 48.3 years). In all cases, the diffuse peritonitis was caused by the gastric ulcer perforation. The control group (CG) consisted of 15 persons, including 11 women and four men. The women’s age ranged from 24 to 77 years (mean age of 49.3 years), whereas men’s age ranged between 35 and 73 years (mean age of 59.5 years). Conditions other than acute abdominal diseases were the reason for hospitalization in these cases.

**Fig. 1:**
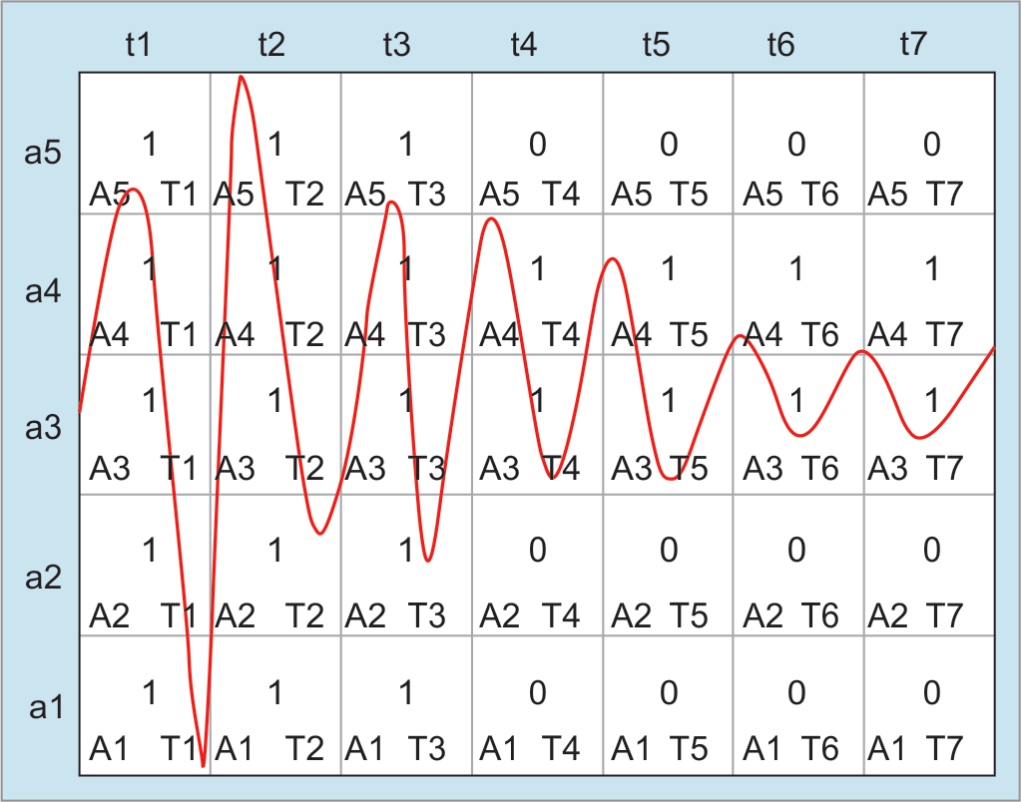
A five-by-seven adjustable grid superimposed on sound impulse

The recorded sound files were subjected to preliminary analysis using commonly available digital audio editors. In the second stage of the study, the method of adjustable grids^11-13^ was utilized for the computer analysis of recordings. It was originally developed for speech recognition and consisted of superimposing two-dimensional grids on sound impulses, whose location and width were determined based on signal local minima. The grid width was automatically adjusted to sound duration and the grid height to its amplitude. Next, the grid cells crossed by the signal were filled with ones and the cells without signal with zeros. Thus, the binary matrix was obtained, which can be further analyzed by comparing it with standard matrices stored in a database in order to identify disease entities (Fig. 1).

The algorithm consisted of the following steps: (1) determining the reference level for local minima by averaging the values of the first 100 signal samples, (2) reading consecutive samples and saving the start and stop times of sound impulses, (3) determining the number of sound impulses in the recording, (4) combining adjacent sound impulses (interval < 0.01 s), (5) superimposing adjustable grids of 35 cells (5 × 7) on combined impulses, (6) calculating a binary matrix for each combined impulse, (7) finding similar matrices in a database, (8) calculating mean bit-similarity between matrices, (9) determining the minimum, maximum and the mean time intervals between the impulses. The recordings of 60 subjects from all groups were analyzed using this algorithm. The following continuous variables were obtained directly from this analysis: number of sound impulses (NSI), number of combined sound impulses (NCI), mean bit-similarity (MBS) expressed as a percentage, minimum (TMIN), maximum (TMAX) and mean (TMEAN) time between impulses expressed in seconds.

In order to explicitly characterize the position within a matrix, its coordinates, e.g. A1_T1, were determined (Table 1). Three methods were used to analyze the matrices. The first one consisted in the statistical verification of variables describing matrices, which were derived from the calculation of the number of bits equal to one in the matrix rows and columns (Table 2). The TIME_SC variable was introduced to distinguish bits equal to one in the matrix columns. As a result, the bits equal to one in column 7 had 7 times higher value than those in column 1. An analogous method was applied to rows (the SEG_SC variable). The structure of the whole matrix was reflected in the TOTAL variable taking into account the presence of 1’s in each column and row simultaneously.

Next, the matrices for patients within groups were compared to determine the number of matched bits (pairs 1-1 and 0-0) at the same positions in both matrices and the number of differing bits (pairs 1-0 and 0-1). The variables shown in Table 2 were used for statistical analysis. Finally, the relationships between the presence of 1 or 0 at a given position in the matrix and the occurrence of diseases were verified. They were analyzed at each of the 35 positions in the matrix for all groups together and for pairs of groups.

The results were subjected to statistical analysis. The normal distribution of continuous variables was verified with the Shapiro-Wilk test. Non-parametric tests (Kruskal-Wallis ANOVA and post hoc tests) were used for comparisons due to the lack of normal distribution and variance homogeneity as well as low sample sizes. The analysis of categorical variables (zero or one in the matrix and disease entity) was based on contingency tables using the Chi-square and Fisher’s exact tests.^14^ The differences were considered statistically significant at p ≤ 0.05.

**Table 1:** **Table 1:** The way of defining positions in the matrix

*Row/column*		*t1*		*t2*		*t3*		*t4*		*t5*		*t6*		*t7*	
a5		A5_T1		A5_T2		A5_T3		A5_T4		A5_T5		A5_T6		A5_T7	
a4		A4_T1		A4_T2		A4_T3		A4_T4		A4_T5		A4_T6		A4_T7	
a3		A3_T1		A3_T2		A3_T3		A3_T4		A3_T5		A3_T6		A3_T7	
a2		A2_T1		A2_T2		A2_T3		A2_T4		A2_T5		A2_T6		A2_T7	
a1		A1_T1		A1_T2		A1_T3		A1_T4		A1_T5		A1_T6		A1_T7	

**Table 2:** **Table 2:** Variables describing binary matrices

*Variables*				*Description*	
TIME_SC				Matrix position calculated according to the formula: 7 × T7 + 6 × T6 + 5 × T5 + 4 × T4 + 3 × T3 + 2 × T2 + T1, where T1, ... ,T7 are the sums of bits equal to 1 in columns t1, ... ,t7	
SEG_SC				Matrix position calculated according to the formula: 5 × A5 + 4 × A4 + 3 × A3 + 2 × A2 + A1, where A1, ... , A5 are the sums of bits equal to 1 in rows a1, ... , a5	
IS_1				Percentage of bits equal to 1 in the matrix	
IS_1_A5 - IS_1_A1				Percentage of bits equal to 1 in rows a5 - a1	
IS_1_T1 - IS_1_T7				Percentage of bits equal to 1 in columns t1 - t7	
TOTAL				The sum of products of coordinates for bits equal to 1 (Σ a1 ... 5 × t1 ... 7)	
P_1_1				Number of compatible 1-1 pairs	
P_1_1P				Percentage of compatible 1-1 pairs	
P_1_0				Number of incompatible 0-1 or 1-0 pairs	
P_1_0P				Percentage of incompatible 0-1 or 1-0 pairs	
P_0_0				Number of compatible 0-0 pairs	
P_0_0P				Percentage of compatible 0-0 pairs	
P_00_11				Number of compatible 0-0 or 1-1 pairs (sum)	
P_00_11P				Percentage of compatible 0-0 or 1-1 pairs	

## RESULTS

The analysis of variables describing BS revealed that the number of sound impulses was significantly lower in the IL and PE patients compared with the CG. For the remaining variables, no statistically significant differences were found (Table 3).

The correlation analysis of these variables showed a statistically significant negative relationship between the number of impulses and the maximum time interval between impulses in the IL group (Kendall’s tau coefficient equal to -0.538) and the APP group (Kendall’s tau coefficient equal to -0.358).

**Table 3:** **Table 3:** Mean values of variables describing bowel sounds (standard deviations in parentheses)

*Variables*		*CG* *(n = 15)*		*APP* *(n = 27)*		*IL* *(n = 14)*		*PE* *(n = 4)*	
NSI		2179.3^a^		1764.9		1004.4^b^		466.3^b^	
		(1487.4)		(1479.4)		(1118.0)		(251.7)	
NCI		17.1 (19.0)		13.1 (12.1)		8.5 (4.1)		12.8 (8.2)	
MBS (%)		53.7 (6.5)		59.0 (13.5)		57.7 (9.1)		57.7 (6.1)	
TMIN (s)		0.1 (0.1)		0.1 (0.2)		0.6 (1.4)		0.1 (0.1)	
TMAX (s)		2.9 (3.2)		4.1 (5.6)		7.5 (9.3)		20.5 (36.0)	
TMEAN (s)		0.7 (0.6)		1.2 (1.2)		2.8 (3.6)		3.6 (6.0)	

^a,b^Mean values in the same row with different superscripts differ significantly (p ≤ 0.05)

**Table 4:** **Table 4:** Mean values of variables describing binary matrices (standard deviations in parentheses)

*Variables*		*CG (n = 15)*		*APP (n = 27)*		*IL (n = 14)*		*PE (n = 4)*	
TOTAL		162.7 (84.2)		166.0 (61.2)		179.4 (74.7)		157.5 (53.3)	
SEG_SC		41.1 (23.1)		41.7 (14.9)		45.7 (18.1)		39.8 (7.0)	
TIME_SC		54.5 (28.1)		51.0 (22.9)		57.7 (25.4)		41.8 (20.4)	
IS_1		41.1 (22.4)		37.0 (16.7)		41.4 (18.8)		29.3 (11.3)	
IS_1_A1		47.6^a^ (27.4)		32.3 (18.8)		38.8^a^ (17.2)		14.3^b^ (16.5)	
IS_1_A2		42.9 (36.2)		30.2 (31.3)		35.7 (31.1)		10.7 (21.4)	
IS_1_A3		43.8 (39.1)		37.6 (33.7)		41.8 (40.2)		21.4 (24.7)	
IS_1_A4		35.2 (33.7)		36.0 (25.8)		36.7 (30.5)		32.1 (24.4)	
IS_1_A5		36.2^a^ (18.6)		49.2 (19.9)		54.1 (25.2)		67.9^b^ (24.4)	
IS_1_T1		45.3 (35.0)		34.9 (26.3)		34.3 (24.1)		30.0 (11.5)	
IS_1_T2		52.0 (29.1)		38.5 (24.1)		47.1 (27.9)		25.0 (10.0)	
IS_1_T3		41.3 (26.7)		38.5 (19.2)		40.0 (19.2)		30.0 (20.0)	
IS_1_T4		40.0 (25.1)		41.5 (22.1)		45.7 (27.7)		30.0 (11.6)	
IS_1_T5		37.4 (24.9)		34.8 (22.6)		47.1 (31.0)		25.0 (10.0)	
IS_1_T6		34.7 (20.7)		31.1 (16.0)		42.9 (23.4)		35.0 (19.2)	
IS_1_T7		37.3 (21.2)		37.8 (23.8)		32.9 (18.6)		30.0 (25.8)	

^a,b^ Mean values in the same row with different superscripts differ significantly (p ≤ 0.05)

**Table 5:** **Table 5:** Mean values of variables describing examined groups in terms of bit-similarity (standard deviations in parentheses)

*Variables*		*CG (n = 15)*		*APP (n = 27)*		*IL (n = 14)*		*PE (n = 4)*	
N*		105		351		91		6	
P_1_1		6.1 (5.2)		4.9 (3.5)		5.8 (4.2)		4.0 (0.9)	
P_1_0		16.5 (5.9)		16.1 (5.1)		17.4 (5.1)^a^		12.5 (5.5)^b^	
P_0_0		12.3 (6.7)^a^		13.1 (6.1)^b^		11.8 (6.1)^a^		18.5 (4.9)^b^	
P_00_11		18.5 (5.9)		18.9 (5.7)		17.7 (6.0)^a^		22.5 (5.5)^b^	

^a,b^Mean values in the same row with different superscripts differ significantly (p ≤ 0.05)

*Number of comparisons within a group

In the analysis of variables describing binary matrices, a significantly lower percentage of bits equal to one in the a1 row were found in the PE patients compared with the IL patients and the CG (Table 4). On the other hand, the percentage of bits equal to one in the a5 row was significantly higher in the PE group compared with the CG.

In the analysis of variables describing bit-similarity, a significantly higher number of differing pairs (0-1 or 1-0) was found in the IL group compared with the PE group (Table 5). Also, significant differences were observed in the number of 0-0 pairs between the CG and the APP and PE groups. In addition, a significantly lower number of 0-0 pairs were recorded in the IL group compared with the APP and PE groups. Finally, a significant difference was observed in the number of 0-0 or 1-1 pairs between the IL and PE groups.

The last stage of the present work was the verification of relationships between the presence of 1 or 0 at a given position in the matrix and the patient’s group (APP, IL, PE or CG). The A1_T1 and A1_T2 positions were associated with IL, whereas the A1_T2 and A1_T4 positions were related to APP. For PE, significant positions in the binary matrices included A5_T4 and A1_T4.

## DISCUSSION

The auscultation of the abdomen has been applied in clinical practice for centuries. It is a simple, non-invasive and safe method.^15^ However, it lacks objectivity and makes it impossible to compare the results of different examinations. The first attempt at making it more objective was made by Cannon^16^ in 1905, who presented his own BS graphically. In the present study, the electronic system was used. As shown by Craine et al,^17^ the use of an electronic stethoscope and digital analysis allows for the detection of approximately 5 times more sounds than during the standard examination. Besides, this method enables an objective and quantitative evaluation of BS.^15,17-22^

One of the basic problems with BS recording is the occurrence of noise, which partially results from the friction between the microphone and the skin during the patient’s respiratory movements.^23^ Various solutions have been proposed to eliminate such artefacts.^17,20-22,24^ In the present study, the shape of the sleeve used with the microphone ensured good adhesion to the abdominal wall and the possibility of recording the signal from an area of 12.5 cm^2^. The next source of potential noise is the surroundings where the examination is performed. The best method for eliminating such unnecessary sounds is recording in a soundproof chamber.^21,22^

Regardless of the application of the above-mentioned methods, the denoising stage is crucial to an appropriate BS analysis.^17,24-26^ Various algorithms based on wavelets and filters were proved to be successful in this respect.^25,27,28^ According to Yoshino et al^22^ noise can also be generated by the sounds accompanying the heart-beat and respiratory function. The preliminary analysis of the sound files in the present study showed that when the microphone was located on the right side of the hypogastrium, these impulses were so weak that they did not exceed the background level. Therefore, they were not analyzed by the program used in the present work. A similar opinion was presented by Craine et al.^17^

The time of BS recording was empirically set at 4 minutes. This choice was mainly dictated by the necessity of performing quick diagnosis in a patient requiring surgical intervention. At the same time, sound duration is sufficient to carry out further analysis. Yoshino, like Sugrue and Dalle, used 10 to 15-minute recordings, however, they did not explain their choice.^20-22^ Craine et al^17^ examining patients with the irritable colon syndrome recorded BS for 2 minutes, whereas Kelemen^29^ considered it essential to auscultate BS for at least 5 minutes.

The problem of the saturation effect present in the BS recordings remained unsolved in the present study. According to Horn and Mynors,^23^ BS are characterized by low energy observed on the surface of the integuments and by large dynamics, i.e. periodic occurrence of loud sounds. The calibration of the system for sounds of high energy may lead to the loss of quiet impulses. On the other hand, an opposite situation causes the saturation effect, when the impulse of high energy appears. This may lead to the errors in the frequency analysis. Although in the present work this phenomenon occurred in some recordings, the saturation effects were relatively small compared with the signal amplitude and they did not significantly bias the results.

The application of adjustable grid method to the BS analysis was motivated by its two main features: (1) it is easy to use and enables rapid examination, and (2) it gives objective and repeatable results that can be verified statistically. Therefore, an attempt was made at identifying sounds associated with acute abdominal diseases. An alternative way of BS classification was proposed by Li et al^24^ who calculated selected signal parameters (average absolute value, square root amplitude, shape factor, crest factor and kurtosis value) and developed classification rules based on these parameters.

In the present work, the number of sound impulses was lower in the IL and PE patients compared with the CG (Table 3). It should be emphasized that the lower number of impulses in the first group occurred during the later stage of IL. Diffuse peritonitis caused the largest decrease in the number of impulses, which confirms a well-known fact. The long silence periods (up to 74s in the PE group and up to 28 and 26s in the IL and APP groups, respectively) are noteworthy. However, the analysis of silence duration did not reveal any statistically significant differences. In the study by Ranta et al^27^ the median duration of silence periods between sounds was excluded from further analysis due to the lack of correlation with any of the first four principal components. On the other hand, Sugrue et al^21^ found significant differences in sound intervals between the control group and the patients suffering from appendicitis, acute cholecystitis and obstruction.

The analysis of variables shown in Table 2 was performed in order to describe the distribution of 1’s in the matrices. It was found that there were more bits equal to one in the a5 row in the PE group than those in the CG. An opposite situation occurred in the a1 row of the matrix. In the IL group, a statistically significant larger numbers of bits equal to one in the a1 row were observed compared with the PE group, but this result was uncertain due to the low sample size. In the remaining cases, the differences were not statistically significant (Table 4). These results lead to the conclusion that the characterization of BS using binary matrices may be useful for distinguishing among various disease entities.

The analysis of bit-pairs showed that the highest similarity existed for the 0-0 pairs in the PE patients. This proves the repeatability of zero bits at corresponding positions in the matrices for individual patients of this group. These results were also significantly different from those in the CG (Table 5). The smallest number of differing pairs (0-1 and 1-0) was found in the PE patients indicating the greatest homogeneity of the distribution of 0 and 1 bits in this group. When comparing the number of matched 0 bits between the APP patients and the CG (Table 5), significantly more 0-0 pairs located at corresponding positions in the matrix were found in the APP patients. Summarizing, it should be noticed that the highest similarity of the 0-0 pairs existed in the PE and APP groups, whereas the number of matched 1-1 pairs was similar in all groups.

The analysis of relationships between the presence of one or zero at a given position in the matrix and the membership in a given group made it possible to indicate the key positions in the matrices, where the presence of bits was highly correlated. Matrix positions, where the value of bits (0, 1) was not accidental and depended on the type of disease were determined. In the present work, the relationship existed only at four positions in the matrix with 35 cells. This result did not allow us to develop standard matrices that could be used for disease identification. The application of the method presented in this study in clinical diagnostics requires its further improvement. However, the stages of BS recording and storing were fully developed. The recordings were of high quality and could have been further analyzed.

In summary, the method employed in this study makes it possible to determine the number of sound impulses and silence duration. The results are in accordance with those presented by other authors. The indicated key positions in the matrices, where the statistically significant relationships existed, allow us to conclude that it would be possible to design standard matrices and determine the sounds that are characteristic of a given disease entity. The isolation of pathognomonic sounds would, in turn, allow for the development of a simple diagnostic method based on the auscultation of the abdomen.
